# The Warburg Effect as a Type B Lactic Acidosis in a Patient With Acute Myeloid Leukemia: A Diagnostic Challenge for Clinicians

**DOI:** 10.3389/fonc.2018.00232

**Published:** 2018-06-20

**Authors:** Clément Brault, Yoann Zerbib, Caroline Delette, Julien Marc, Bérengère Gruson, Jean P. Marolleau, Julien Maizel

**Affiliations:** ^1^Réanimation Médicale, CHU Amiens-Picardie, Amiens, France; ^2^Hématologie Clinique, CHU Amiens-Picardie, Amiens, France

**Keywords:** lactic acidosis, hypoglycemia, the Warburg effect, acute leukemia, intensive care unit, chemotherapy

## Abstract

**Introduction:**

The Warburg effect (WE) is an uncommon cause of type B lactic acidosis (LA) due to a deregulation of carbohydrate metabolism in neoplastic cells where lactic fermentation predominates over oxidative phosphorylation regardless of the oxygen level.

**Case presentation:**

We report the case of a 57-year-old man presenting with concomitant acute myeloid leukemia and type B LA with asymptomatic hypoglycemia. We did not find arguments for a septic state, liver dysfunction, or acute mesenteric ischemia. The WE was suspected, and chemotherapy was immediately undertaken. We observed a rapid and sustained decrease in lactate level and normalization of blood glucose. Unfortunately, we noted a relapse of acute leukemia associated with WE soon after treatment initiation and the patient died in the Intensive Care unit.

**Discussion:**

Some patients may present complications directly related to an underlying hematological malignancy. The WE is one of these complications and should be suspected in patients with both hypoglycemia and LA. We propose a checklist in order to help clinicians manage this life-threatening complication. Before considering WE, clinicians should eliminate diagnoses such as septic shock or mesenteric ischemia, which require urgent and specific management.

**Conclusion:**

The diagnosis of WE can be challenging for clinicians in the Hematology department and the Intensive Care unit. Prompt diagnosis and rapid, adapted chemotherapy initiation may benefit patient survival.

## Introduction

Lactic acidosis (LA) is defined as a pH of less than 7.35 and a blood lactate level greater than 5 mmol/L (1). Type A LA is related to inadequate tissue perfusion in a context of sepsis, circulatory failure, hypovolemia, or severe hypoxemia. Conversely, type B LA occurs without any sign of organ dysfunction and is related to a deregulation of cellular metabolism (1–5).

Lactic acidosis is a common and serious complication at the onset or during the management of hematological malignancies, most often related to type A LA. A recent study including 372 patients with hematological malignancies admitted to the Intensive Care unit reported 58 cases of LA, among which only six cases were type B LA (6).

The Warburg effect (WE), or hyper-Warburgism, is a classic but challenging cause of type B LA in hematological malignancies. The WE is due to a specific malignant-cell metabolism, where lactic fermentation predominates over oxidative phosphorylation regardless of the oxygen level (7). It has mostly been associated with patients with lymphoma, but also with those with acute leukemia, chronic lymphocytic leukemia, chronic myelomonocytic leukemia, or multiple myeloma (1, 5, 8). We report the case of a 57-year-old man who developed WE as a consequence of acute myeloid leukemia (AML). We propose a checklist to assist clinicians in the management of this life-threatening complication.

## Case Presentation

A 57-year-old man presented with neutropenia, since May 2016 due to a myelodysplastic syndrome. The revised international prognostic scoring system was 0, and no specific treatment was undertaken. Other significant past medical history included well-controlled hypertension treated with quinapril and type 2 diabetes mellitus without medication.

In November 2016, he experienced severe asthenia and excessive sweating. Laboratory tests revealed leukocyte count of 8,000 per cubic millimeter with hyperblastosis (23%), anemia, and thrombocytopenia. The results of the bone marrow aspiration confirmed AML (M4 type) according to the French–American–British classification, without extramedullary manifestations. FLT3, CEBPα, and NPM1 were not mutated and no cytogenetic abnormalities were found. This AML was secondary to a myelodysplastic syndrome with single lineage dysplasia. For these reasons, the patient was eligible for a hematopoietic stem cell allograft.

In the Hematology department, an asymptomatic hypoglycemia that persists despite glucose infusion was found. Laboratory tests showed type B LA with an elevated blood lactate of 14 mmol/L (normal range, 0.5–2 mmol/L) associated with a slightly decreased pH of 7.35 (normal range, 7.38–7.42). Serum bicarbonate was low at 13 mmol/L (normal range, 24–32 mmol/L) with normal renal function tests and an elevated anion gap of 28 mmol/L. Liver function tests were normal.

**Figure 1:**
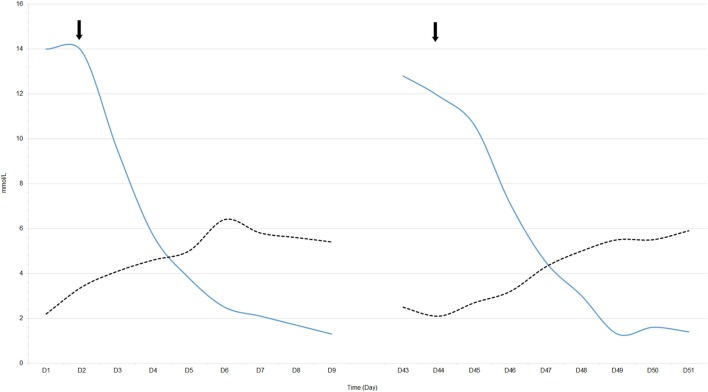
Blood lactate and glucose levels following chemotherapy initiation in our patient at admission (D1) and after relapse (D43). ↓ Chemotherapy administration, solid line: blood lactate level, dotted line: blood glucose level.

The patient was transferred to the Intensive Care unit. His temperature was 37.7°C, his blood pressure was normal at 149/82 mmHg, his pulse was 119 bpm, and the respiratory rate was 28 per minute without respiratory distress which indicated Kussmaul breathing. The patient did not present any signs of hypoperfusion (he had normal blood pressure, absence of mottling, normal capillary refilling test). The palpation of the abdomen was normal without diarrhea. In the absence of a type A LA etiology, and in the context of AML, we suspected WE. Chemotherapy by doxorubicin 60 mg/m^2^ and cytarabine 100 mg/m^2^ was initiated at day 1. A slight tumor lysis syndrome was observed without kidney injury. We noted no recurrence of hypoglycemia, a rapid normalization of pH, and a decrease in the blood lactate level: 12.1 mmol/L at day 2, 9.2 mmol/L at day 3, 2.6 mmol/L at day 5, and 1.6 mmol/L at day 7 (Figure 1). We observed no further complications, allowing ICU discharge at day 8.

Unfortunately, a relapse of AL occurred at day 43. This relapse was associated with a new hyperlactatemia (pH: 7.37 and blood lactate level: 12.8 mmol/L) and asymptomatic hypoglycemia related to a recurrence of WE (Figure 1). Salvage chemotherapy with cytarabine 1,500 mg/m^2^ and gemtuzumab 3 mg/m^2^ was initiated, resulting in the disappearance of WE. However, the patient later developed septic shock as a complication of chemotherapy-induced aplasia and digestive infection. We suspected infectious colitis because of diarrhea and abdominal guarding. Unfortunately, the CT scan was not contributory and we could not perform a colonoscopy to confirm the diagnosis. Empirical broad-spectrum antibiotic therapy with imipenem, vancomycin, and gentamycin was administered. He eventually died in the Intensive Care unit in a state of multiple organ dysfunction due to the septic shock.

## Discussion

Here, we reported the case of an AML complicated by a WE, an uncommon cause of type-B LA. In aerobic conditions, oxidative phosphorylation by the mitochondria is the main source of cellular energy, with the delivery of 38 units of adenosine triphosphate (ATP) per molecule of glucose. In anaerobic conditions, the mitochondrial respiratory chain complex is non-functional, so the energy supply is provided by lactic fermentation, with the delivery of only two units of ATP per molecule of glucose and some degree of lactate accumulation. The WE is a specific malignant-cell metabolism (in hematological malignancies and solid neoplasms) where lactic fermentation predominates over oxidative phosphorylation, regardless of the oxygen level (9, 10). Mechanisms explaining WE in neoplastic cells are misunderstood, but depend on the microenvironmental supply of oxygen, overexpression of factors promoting the lactic fermentation pathway (e.g., hexokinase, insulin-like growth factors, or hypoxia-inducible factor 1α), or inflammatory cytokines (e.g., tumor necrosis factor alpha) (10, 11). Recently, oncogenes like FLT3 tyrosine kinase, K-ras, c-Myc, and Bcr-Abl have been shown to induce WE promoting glycolysis or mitochondrial dysfunction (12). However, the search for the mutation in FLT3 tyrosine kinase, usually detected in 30% of AML, was negative in our patient. The WE, combined with the rapid growth and replication of tumor cells and impairment of lactate clearance by the liver or kidneys, can explain blood lactate accumulation and occurrence of metabolic acidosis (6).

**Figure 2:**
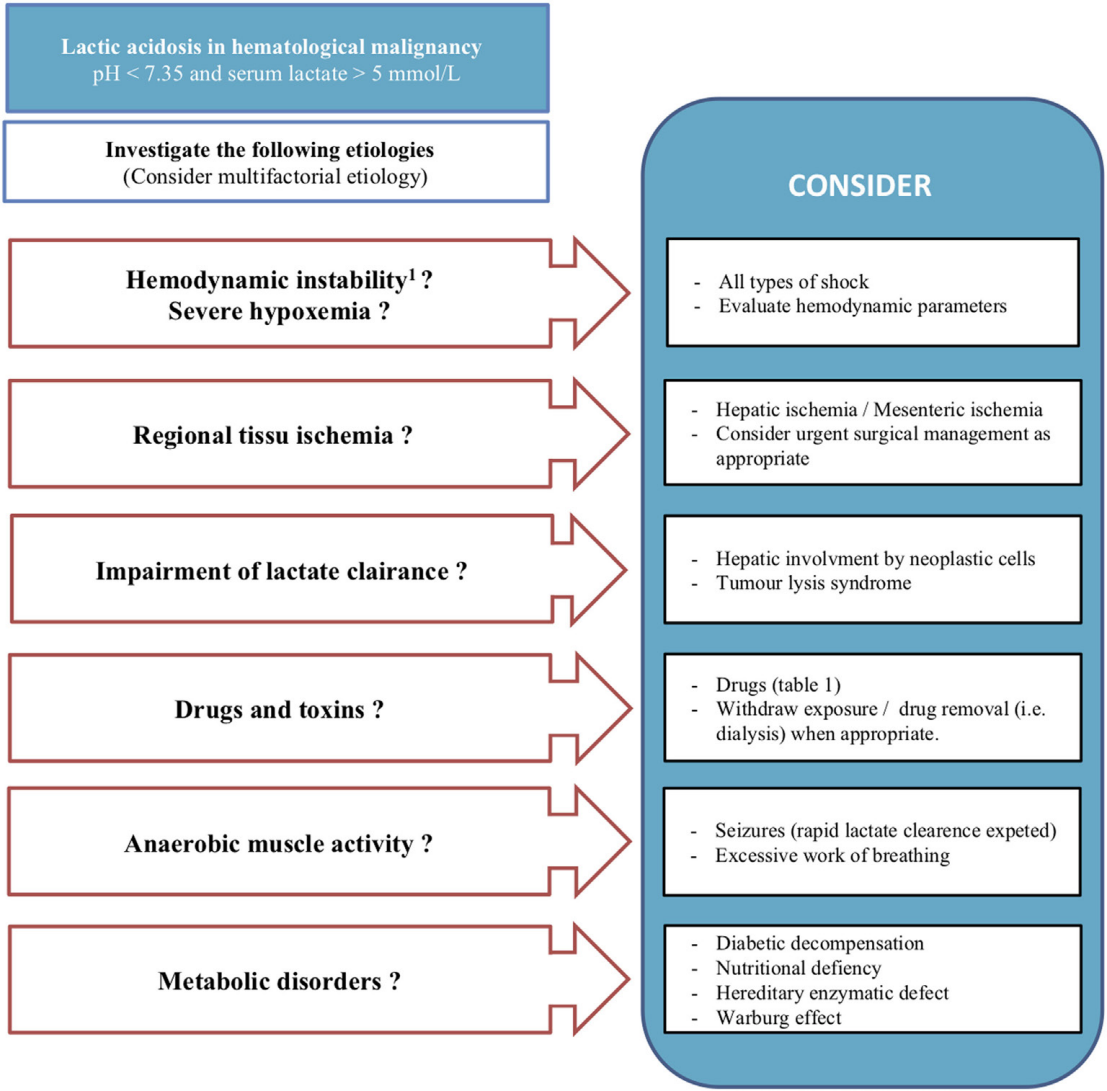
LA in hematological malignancy: a checklist for clinicians. Mechanisms responsible for LA are not mutually exclusive and can co-exist in the same patient. ^1^Mean arterial blood pressure <65 mmHg or use of vasoactive agent, mottling, or prolonged capillary refilling test. LA, lactic acidosis.

In the presence of LA in patients with hematological malignancies, clinicians should evoke different etiologies. It should be borne in mind that mechanisms responsible for LA in patients with malignancies are not mutually exclusive and can co-exist (Figure 2).

Classic causes are hemodynamic instability associated with hypoperfusion (mean arterial blood pressure lower than 65 mmHg or use of catecholamine, mottling, prolonged capillary refilling time) or severe hypoxemia. Many etiologists of hemodynamic instability exist, but particular attention should be paid to infections (especially pneumonia or gastro-intestinal infections) in these immunocompromised patients (13).

Regional tissue ischemia can also be responsible for LA. The diagnosis of mesenteric ischemia should be considered, keeping in mind that performance of clinical and radiological signs are limited. Endoscopic explorations of the digestive tract, which can help us to confirm or exclude this diagnosis, are often difficult to carry out because of the high frequency of thrombocytopenia or coagulopathy associated with malignancies. Coral or tumor thrombi, vascular mesenteric infiltration, and extrinsic vascular compression are the main mechanisms leading to mesenteric ischemia (14).

Hepatic infiltration by lymphoma or leukemia can lead to lactate clearance impairment. This involvement is frequent, but rarely causes acute and severe hepatic failure with LA. Liver biopsy is often necessary for diagnosis because of the lack of specificity of radiologic signs. The progression of the disease is often fatal despite chemotherapy (2, 15). Similarly, renal failure due to tumor lysis syndrome is common in hematological malignancies and may contribute, to a lesser extent, to lactate accumulation (16).

Finally, less frequent LA causes can be considered: hyperosmolar hyperglycemic states or other complications of diabetes mellitus, nutritional deficiency (e.g., thiamine deficiency), anaerobic muscle activity, and drugs or toxins. Among drugs implicated, metformin or nucleoside reverse transcriptase inhibitors are the most common (Table 1). More rarely in adult patients, some hereditary enzymatic defects (e.g., glucose-6-phosphatase deficiency) resulting in type B LA have been described (2–5, 17).

**Table 1:** Drugs associated with type B lactic acidosis.

Drugs
Acetaminophen
Antiretroviral therapy (e.g., stavudine, zidovudine, and lamivudine)
β-2 agonists (e.g., salbutamol, terbutaline)
Biguanides (e.g., metformin, phenformin)
Isoniazid
Lactulose
Linezolid
Nalidixic acid
Niacin
Nitroprusside sodium
Propofol
Simvastatin
Theophylline
Vasoactive drugs (e.g., epinephrine, norepinephrine)

A literature review found only five patients with AML associated with type B LA (5, 18–21). Among them, lactate clearance impairment was suspected in three patients because of a history of liver cirrhosis (5), and a possible liver infiltration by leukemia cells as well as severe renal failure were also found (18, 19). Thiamine deficiency was proved in one patient (20). This illustrates that several mechanisms leading to hyperlactatemia can co-exist in the same patient.

Clinical examination is nonspecific in WE. Tachypnea without hypoxemia relevant to LA—Kussmaul breathing—is often on the forefront of clinical attention. Severe hypoglycemia is classic and evocative, but not always present. It is completely asymptomatic because lactate is used by the brain as a source of energy by a misunderstood mechanism involving astrocytic and neuronal cells. This specificity explains the absence of fluorodeoxyglucose uptake in the brain in positron emission tomography/computed tomography studies of patients with WE. Hypoglycemia is often not correctable by dextrose infusion (22). Abnormal laboratory findings frequently include a very high blood lactate level (>10 mmol/L), contrasting with the absence of signs of tissue hypoperfusion (6).

Since there is no randomized controlled trial, we can hardly provide any specific and definite recommendations for WE management. Nevertheless, a fast diagnostic workup of WE and the understanding of the implication of the underlying malignancy in the metabolic disorder seems to be essential. This implies that chemotherapy administration is the main therapeutic action for fast recovery, with the aim of disrupting lactate production by tumor cells (1). Regression of hyperlactatemia is the general rule and is a good retrospective argument for WE diagnosis (21).

Other therapeutic options can be discussed, but lack solid and relevant arguments. Renal replacement therapy has been initiated in several cases with no effect on the lactate level, probably because of inadequate lactate clearance compared to lactate production (1, 23). Thus, the impact of renal replacement therapy on WE remains questionable. Sodium bicarbonate infusion has also been used for its proton binding property, but we can hardly support this strategy because of potential complications (e.g., intracellular acidification, volume overload) (23). In addition, some authors have tested empirical thiamine infusion for the potential benefit for mitochondrial function without severe risks (1). Thiamine deficiency could occur in patients with malignancies, explained by exclusive parenteral nutrition without thiamine supplementation (5). Moreover, some chemotherapy (e.g., methotrexate) may limit the cellular bioavailability of thiamine (24). Thiamine plays a key role as a pyruvate dehydrogenase enzyme cofactor, promoting the aerobic glycosylation pathway (25). Therefore, we recommend routine thiamine supplementation.

In our experience, WE occurs when there is a high tumor burden with a high proliferation rate, often complicated by an induced tumor lysis syndrome (26, 27). Therefore, the presence of WE in a patient receiving hematological care should be considered as having a major risk factor for tumor lysis syndrome and should lead clinicians to initiate prophylactic treatment. Patient survival is determined by the body’s ability to correct the metabolic acidosis induced by hyperlactatemia. This compensation is mainly related to alveolar hyperventilation. The excessive effort relative to the strength and endurance of the respiratory muscles can induce respiratory failure resulting in a sudden loss of compensation for acidosis. Data on the mortality of WE patients are limited, but type B LA seems to be a poor prognostic factor of survival in case series (6–8).

## Conclusion

Type B LA is a challenging situation for clinicians and can indicate complications of malignancies. The WE is a rare but severe condition that needs to be recognized. Clinicians should suspect WE in patients with hematological malignancies who develop both type B LA and acute asymptomatic hypoglycemia. The management of such specific complications relies on urgent initiation of chemotherapy along with life support management.

## Consent for Publication

Written informed consent was obtained from the next of kin for the publication of this case report.

## Ethics Statement

Ethic approval and consent to participate: according to the French legislation, no ethics approval was necessary. Consent for publication: because of the death of the patient, only the consent of his wife could be obtained.

## Author Contributions

CB and YZ have an equal contribution. CB, YZ and JMaizel wrote the manuscript. CD, BG, JMarc and JPM had a critical review of the manuscript. All authors read and approved the final manuscript.

## Conflict of Interest Statement

The authors declare that the research was conducted in the absence of any commercial or financial relationships that could be construed as a potential conflict of interest.
